# Search of Novel Small Molecule Inhibitors for the Main Protease of SARS-CoV-2

**DOI:** 10.3390/v15020580

**Published:** 2023-02-20

**Authors:** Wenfa Zhang, Sheng-Xiang Lin

**Affiliations:** Axe Molecular Endocrinology and Nephrology, CHU Research Center and Laval University, Québec City, QC G1 V 4G2, Canada

**Keywords:** SARS-CoV-2 main protease, molecular docking, protease kinetics inhibition, ligand/protein interaction, toxicity, inhibitor efficacy

## Abstract

The current outbreak of coronavirus disease 2019 (COVID-19) has prompted the necessity of efficient treatment strategies. The COVID-19 pandemic was caused by the severe acute respiratory syndrome coronavirus 2 (SARS-CoV-2). Main protease (Mpro), also called 3-chymotrypsin-like protease (3CL protease), plays an essential role in cleaving virus polyproteins for the functional replication complex. Therefore, Mpro is a promising drug target for COVID-19 therapy. Through molecular modelling, docking and a protease activity assay, we found four novel inhibitors targeting Mpro with the half maximal inhibitory concentration (IC50) and their binding affinities shown by the dissociation constants (K_D_s). Our new inhibitors CB-21, CB-25, CP-1 and LC24-20 have IC50s at 14.88 µM (95% Confidence Interval (95% CI): 10.35 µM to 20.48 µM), 22.74 µM (95% CI: 13.01 µM to 38.16 µM), 18.54µM (95% CI: 6.54 µM to 36.30 µM) and 32.87µM (95% CI: 18.37 µM to 54.80 µM)), respectively. The evaluation of interactions suggested that each inhibitor has a hydrogen bond or hydrophobic interactions with important residues, including the most essential catalytic residues: His^41^ and Cys^145^. All the four inhibitors have a much higher 50% lethal dose (LD50) compared with the well-known Mpro inhibitor GC376, demonstrating its low toxicity. These four inhibitors can be potential drug candidates for further in vitro and in vivo studies against COVID-19.

## 1. Introduction

The recent outbreak of COVID-19 caused by SARS-CoV-2 has rapidly become a pandemic since its first report in December, 2019 [1]. More than 640 million total cases have been reported, with 6.6 million deaths worldwide by 25 November 2022 (Johns Hopkins Coronavirus Resource Center, https://coronavirus.jhu.edu/map.html (accessed on 25 November 2022)).

SARS-CoV-2 is a single strand positive-sense RNA virus that is highly contagious in humans [2]. It has 10 open reading frames (ORFs): ORF1a, ORF1b, spike protein (S), ORF3a, Envelope protein (E), Membrane (M), ORF6, ORF7a, ORF7b, ORF8, Nucleocapsid (N) and ORF10 [3]. Structural proteins S, E and M together can create the viral envelope, and N is used to hold the RNA genome. ORF1a and ORF1b embed 16 non-structural proteins (nsp1 to nsp16) including the RNA-dependent RNA polymerase (RdRp) used for RNA genome replication and Mpro, and papain-like protease (PLpro) used for polyprotein cleavage, to generate a functional active viral replication complex [4]. The Mpro corresponds to nsp5. PLpro can cleave three conserved sites for functional nsp1, nsp2 and nsp3 and Mpro can cleave the eleven conserved sites for functional nsp4 to nsp16 [5]. Therefore, Mpro is a promising target for drug design. 

Several inhibitors targeting SARS-CoV-2 Mpro have been tested and reported to have a low IC50 or EC50. For example, 11a, 11b, 11r, 13b, or Ebselen have a tested IC50 lower than 1 µM and 13a; Disulfiram, Tideglusib and Carmofur have IC50s between 1 µM to 10 µM, Shikonin has an IC50 at 15.75 µM, PX-12 has an IC50 at 21.39 µM and Cinanserin has an IC50 at 124 µM [6,7,8]. All these inhibitors could be candidate drugs through pre-clinical and clinical tests. According to the data from ClinicalTrial.gov in the USA, several drug candidates targeting Mpro are under evaluation for clinical trial: PBI-0451, Ebselen, Ritonavir, S-217622, Nirmatrelvir, Lopinavir, Apixaban and Azithromycin. However, no drugs have yet been approved by the Food and Drug Administration (FDA). Therefore, searching for a new drug candidate for high efficacy and low toxicity is still necessary.

In this study, we found four novel Mpro inhibitors through docking and validated by in vitro experiments. All the four inhibitors have a relatively high bioavailability score and low toxicity. They thus have low IC50s and low K_D_s, demonstrating a high affinity for Mpro binding. We also evaluated the hydrogen and hydrophobic interactions between each inhibitor and Mpro, demonstrating that each drug has interactions with important amino acid residues from Mpro. These four inhibitors can be potential drug candidates for COVID-19 therapy through preclinical and clinical approvements.

## 2. Materials and Methods

### 2.1. Drug Preparation and Docking Analysis

A total of 26,832 compounds from Life Chemicals (https://lifechemicals.com/ (accessed on 1 October 2021)), 29,524 compounds from Chembridge (https://chembridge.com/ (accessed on 1 January 2022)) and 107,341 compounds from Chemspace (https://chem-space.com/ (accessed on 1 January 2022)) were downloaded for docking analysis. All drugs were converted from a structure data file (.sdf) to mol2 format. Hydrogens were added, and 3D coordinates were also generated at the same time. 

The crystal structure of the main protease (6WTT and 5RGI) was downloaded from the Protein Data Bank (https://www.rcsb.org/ (accessed on 1 October 2021)). The ligand, ions and waters of the crystal structure were removed before docking. GOLD software was used for docking analysis for all compounds. An amount of 10 Å around the original ligand was set as the docking site. For each ligand, a maximum of 10 structures were generated, and the ChemPLP fitness was computed.

### 2.2. IC50 Test

SARS-CoV-2 Mpro and Mpro protease fluorogenic substrate peptide were purchased from Millipore Sigma (Cat#: SAE0172 and SAE0180, respectively). Proteolytic cleavage by Mpro releases the fluorescent AFC group (sequence TSAVLQ) which can be detected by a fluorescence microscope.

IC50 was tested with an 8 ug/mL main protease and a 15 ug/mL substrate in 25 mM HEPES (PH 7.5) in a 96-well plate. The reaction volume was 50 uL. Then 0.5 ul of a different concentration of inhibitors dissolved in 100% DMSO was added to each well and reacted for 90 min. The increase in fluorescence was detected with a microscope (excitation = 400 nm, emission = 505 nm). 

IC50 and the 95% confidence interval (95% CI) was calculated by GraphPad Prism (version 8.0.1).

### 2.3. Dissociation Constant (K_D_) Test

The K_D_ was determined by titration as described previously [9]. A total of 350 ul of mixture with 8 ug/mL main protease, 25 mM HEPES (PH7.5) and 2% DMSO was added to the cuvette. An amount of 2 uL 200 µM of each compound with 25mM HEPES (PH7.5) and 2% DMSO was added into each cuvette and left to wait for 90 s. The data was collected with a fluorometer every 90 s (excitation = 285nm and slit = 2 nm; emission = 480 nm and slit = 8 nm). The affinity of each ligand (*K*_D_) was derived by a linear relation using the Scatchard plot [10]:

Δ*F* = −*K*_D_ Δ *F*/[S] + Δ *F*_∞_

where Δ*F* and Δ *F*_∞_ stands for the fluorescence change at the substrate concentration [S], and the fluorescence change when all enzyme molecules are complexed with the substrate. 

The inner-filter correction was made as below [11]:

$$f=\frac{I_{0}}{I}=\frac{P_{0}+\Delta A}{P_{0}}\frac{1-10^{-P_{0}}}{1-10^{-\left(P_{0}+\Delta A\right)}}$$

where *f*, *I*_0_ and *I* refer to the correction factor, corrected and observed fluorescence intensity, respectively. *P*_0_ and ΔA stands for the sample absorption before titration and absorption change with the addition of substrates. 

### 2.4. Compound Pharmacokinetic, Drug Likeness and Toxicity Predictions

SwissADME (http://www.swissadme.ch/index.php# (accessed on 1 September 2022)) allows one to compute the ADME (Absorption, Distribution, Metabolism, Excretion) parameters, pharmacokinetic properties and drug likeness and medicinal chemistry friendliness [12]. ProTox-II (https://tox-new.charite.de/protox_II/ (accessed on 1 September 2022)) is also a free web server based on a total of 33 models for the prediction of various toxicity endpoints like acute toxicity, cytotoxicity and hepatotoxicity. All compounds were subjected to the SwissADME and ProTox-II for evaluation [13].

### 2.5. Hydrogen and Hydrophobic Interactions Analysis

Interactions between each compound and Mpro were analyzed by LigPlus (version 2.2.5) by default parameters. The maximum distance of the hydrogen–acceptor distance is 2.7 Å, and the donor–acceptor distance is 3.35 Å. For hydrophobic contacts, the minimum contact distance is 2.90 Å, and the maximum contact distance is 3.90 Å.

## 3. Results

### 3.1. Molecular Docking

**Figure 1:**
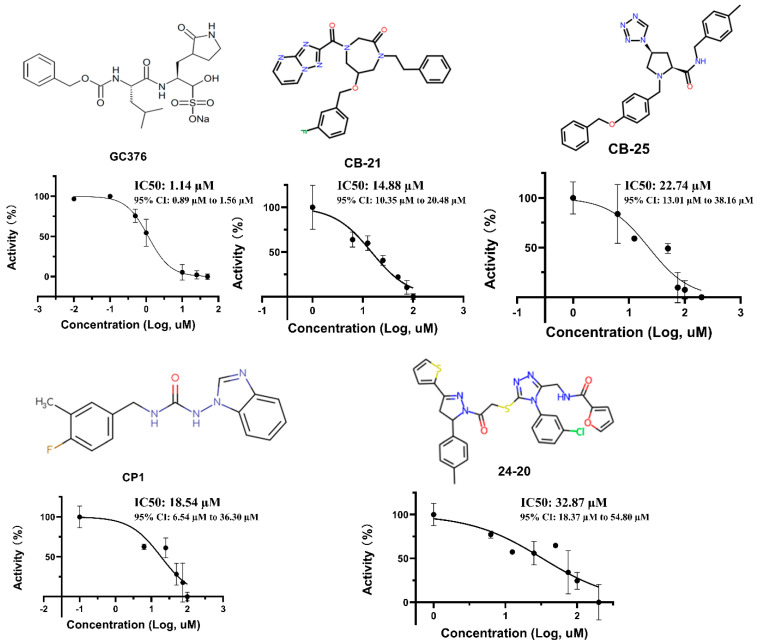
Structure and IC50 test of each compound. 95% CI: 95% Confidence Interval.

An in silico molecular docking method was used to investigate potential drug candidates. A total of 26,832 compounds from Life Chemicals, 29,524 compounds from Chembridge and 107,341 compounds from Chemspace were downloaded for docking analysis. Docking analysis was performed by Genetic Optimization for Ligand Docking (GOLD), which is based on a genetic algorithm for docking flexible ligands. The ChemPLP was utilized as the main scoring function. ChemPLP has been validated as the most effective scoring function using the ChemScore hydrogen bonding term as well as multiple linear potentials to model van der Waals and repulsive terms. The Mpro crystal structure 5RGI was downloaded from the Protein Data Bank, and the ligand Z369936976 was removed for docking. Top ranking drugs were purchased and further tested, and several selected drugs are shown in Table 1 and Figure 1. We found that the docking ChemPLP fitness scores of CB-21 and CB-25 from Chembridge, CP-1 from Chemspace and LC24-20 from Life Chemicals are 98.08, 92.36, 81.19 and 90.29, respectively. These scores demonstrate that the four inhibitors have good potential to inhibit Mpro.

### 3.2. IC50 and Inhibitor Binding

The half maximal inhibitory concentrations (IC50s) of five inhibitors were tested by an enzymatic assay (Figure 1). As the IC50 of the inhibitor Z369936976 in 5RGI was not tested, the well-known SARS-CoV-2 Mpro inhibitor GC376 was used as the positive control in the IC50 test. The GC376 has already been shown to be an effective Mpro inhibitor, with an IC50 ranging from 0.15 µM to 29.4 µM from different reports [14,15,16,17,18]. According to our experiment, the IC50 of GC376 is 1.14 µM (95% CI: 0.89 µM to 1.56 µM), similar to previously reported results. Our newly found inhibitor CB-21 has an IC50 at 14.88 µM (95% CI: 10.35 µM to 20.48 µM) and CB-25 has an IC50 at 22.74 µM (95% CI: 13.01 µM to 38.16 µM). CP-1 has an IC50 at 18.54 µM (95% CI: 6.54 µM to 36.30 µM) and LC24-20 has a higher IC50 at 32.87µM (95% CI: 18.37 µM to 54.80 µM).

The binding of inhibitors to the main protease was parallelly tested by the direct inhibitor titration, yielding the intrinsic fluorescence modification. We found that the K_D_ of CB-21, CB-25, CP-1 and LC24-20 are 12.85 ± 2.05 µM, 10.77 ± 1.32 µM, 5.08 ± 0.38 µM and 20.05 ± 1.27 µM, respectively. Like the IC50 test experiment, the K_D_ of CB-21, CB-25 and CP-1 is lower than the K_D_ of LC24-20.

### 3.3. Drug Likeliness, Pharmacokinetic and Oral Toxicity Evaluations of Selected Compounds 

Drug likeliness, pharmacokinetic and oral toxicity were evaluated for the four inhibitors, as well as the GC376 (Table 2) by SwissADME and ProTox-II. Results show that compared with GC376, all four drugs have high lipophilicity. LC24-20 has the highest lipophilicity, and it is insoluble in water whereas GC376 is soluble in water. CB-21, CB-25 and CP-1 have high gastrointestinal absorption but GC376 and LC24-20 have low gastrointestinal absorption. For the five molecules, only CP-1 has the blood brain barrier permeability. P-glycoprotein is an important protein of cell membrane, primarily expressed in the liver, pancreas, kidney, colon and jejunum [19], and it plays an important role in compound transport. GC376, CB-21 and CB-25 may behave better in their transport. The bioavailability score which refers to the drug becoming completely available to its intended biological destinations of the four drugs is 0.55 and much higher compared with GC376, whose bioavailability score is only 0.17. Similarly, the LD_50_s of CB-21, CB-25, CP-1 and LC24-20 are also much higher than GC376. The LD50 of GC376 is 300 mg/kg whereas the LD50s of CB-21, CB-25, CP-1 and LC24-20 are 1600 mg/kg, 576 mg/kg, 1000 mg/kg and 3000 mg/kg, respectively (Table 2).

### 3.4. Interactions

The main protease has three domains: domain I (residues 8–101), domain II (residues 102–184) and domain III (residues 201–303). Domain I and domain II have an antiparallel β-barrel structure similar to structures of the trypsin-like serine proteases, and domain III consists of five α-helices, forming an antiparallel conglomerate connected to the domain by a long loop region composed of residues 185–200 [20,21]. The active site of SARS-COV-2 Mpro is located in a cleft between domains I and II, holding a histidine/cysteine catalytic dyad (His^41^ and Cys^145^).

**Figure 2:**
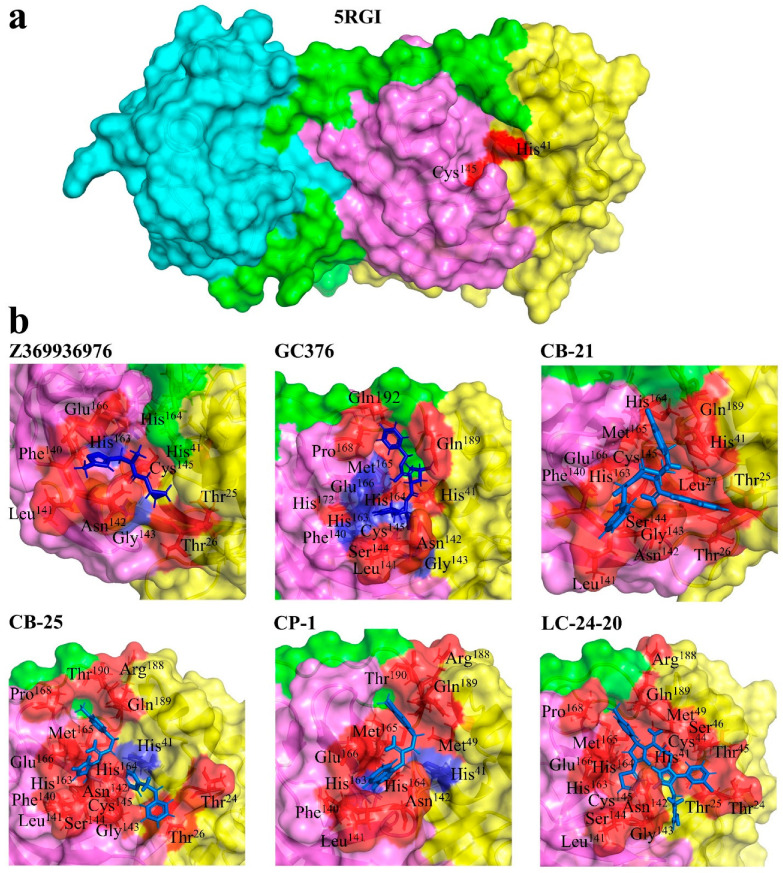
Structure of main protease and interactions with each compound. Main protease has three domains. Domain I (residues 8–101) is colored in yellow, domain II (residues 102–184) is colored in violet and domain III (residues 201–303) is colored in cyan. (**a**) Surface plot of crystal structure 5RGI. Essential catalytic residues His^41^ and Cys^145^ are colored in red. (**b**) Interactions between each inhibitor and main protease. Crystal structure of Mpro with inhibitor GC376 was retrieved from 6WTT and others are retrieved from 5RGI. Residues which have hydrogen bond interactions with ligands are colored in blue while residues which have hydrophobic interactions with ligands are colored in red and shown by sticks.

Elaborating on the interactions between the ligand and the enzyme is important for understanding the enzyme mechanism and developing novel chemical agents or inhibitors [22]. For each inhibitor, the hydrogen bond interactions and hydrophobic interactions between each inhibitor and Mpro were analyzed (Figure 2, Table 3). The amino acid alignment reveals that all residues potentially interacting with the ligand in the active site of SARS-CoV-1 and SARS-CoV-2 are conserved. These residues include Thr^24^, Thr^25^, His^41^, Cys^44^, Met^49^, Tyr^54^, Phe^140^, Asn^142^, Gly^143^, Cys^145^, His^163^, His^164^, Met^165^, Glu^166^, Leu^167^, Pro^168^, Asp^187^, Arg^188^, Gln^189^ and Thr^190^ [21]. All the inhibitors have interactions with the most important catalytic residues His^41^ and Cys^145^. Our results showed that Z369936976 has two hydrogen bond interactions with Mpro: Gly^143^ and His^163,^ while it has nine hydrophobic interactions with Mpro: Thr^25^, Thr^26^, His^41^, Phe^140^, Leu^141^, Asn^142^, Cys^145^, His^164^ and Glu^166^. GC376 has hydrogen bond interactions with five amino acids: Phe^140^, Gly^143^, Cys^145^, His^163^ and Glu^166^ and hydrophobic interactions with another ten amino acids including His^41^. CB-21 has interactions with all residues that interact with Z369936976, and it also interacts with several other residues such as Leu^27^, Ser^144^, Met^165^ and Gln^189^. CB-25 has nine of the same interactions with Z369936976, LC24-20 has eight of the same interactions with Z369936976 and CP-1 has seven of the same interactions with Z369936976. CB-25 only has one hydrogen bond interaction with His^41^ and CP-1 has two hydrogen bond interactions with His^41^ and His^163,^ and all the remaining interactions are hydrophobic interactions.

## 4. Discussion

The ongoing COVID-19 pandemic brought disaster to health and economic crises to people all over the world. Despite the usage of various kinds of vaccines, the pandemic is still spreading worldwide due to the fast viral mutation. As the main protease of SARS-CoV-2, Mpro plays an important role in SARS-CoV-2 replication and thus becomes a promising drug target. 

A total of 163,697 compounds from Life Chemicals, Chembridge and Chemspace were prepared for the docking analysis. The larger the compound number utilized for docking, the more possibility there was to find efficient inhibitors. Compounds with high PLP fitness scores were then tested by an enzymatic assay for IC50. However, a higher PLP fitness score does not always correlate with a lower IC50 for a compound. For example, for the four inhibitors described here, CP-1 has a PLP fitness score of 81.19 and an IC50 at 18.54 uM while LC24-20 has a PLP fitness score of 90.29 and an IC50 at 32.87 uM. In fact most compounds we purchased with a high docking score may have low or even no inhibition to the enzyme. Therefore, more validation methods need to be used jointly for a better prediction. Molecular dynamics-based methods, although computationally expensive, are reported to perform better in ranking molecules compared with docking methods including ChemPLP, AutoDock Vina and Glide [23]. For example, top ranking molecules selected by a PLP fitness score could be further screened by molecular dynamics-based methods before inhibitor purchasing for experimental verification. 

CB-21 has a PLP fitness score of 98.08 and an IC50 at 14.88 uM, seeming to be the best of the four inhibitors found. Compared with the well-known Mpro inhibitor GC376 with a tested IC50 at 1.14 uM (95% CI: 0.89 µM to 1.56 µM) the inhibition efficacy is not high enough. However, CB-21 has a high GI absorption, high bioavailability score and much higher LD_50_ (1600 mg/kg) than GC376. The calculated LD_50_ of inhibitor 11a, 11b and 11r [8], which has an IC50 at less than 1 uM, are 200 mg/kg, 200 mg/kg and 1000 mg/kg, respectively. It is also higher than that of CB-21. Several inhibitors targeting Mpro are under evaluation for clinical trial by the FDA: PBI-0451, Ebselen, Ritonavir, S-217622, Nirmatrelvir, Lopinavir, Apixaban and Azithromycin. Paxlovid (nirmatrelvir and ritonavir) has been issued for emergency use for the treatment of mild-to-moderate cases in certain adults and pediatric patients (Paxlovid LOA 10272022 (fda.gov)). However, no drugs targeting Mpro have yet been approved by the FDA. All of our four new inhibitors were directly purchased from the company and tested by an enzymatic assay without any modification. Proper group modification of the molecular may increase their performance. 

Elaborating on the detailed interactions between the enzyme and ligands is the basis for understanding the binding and catalytic process, being important for developing novel inhibitors or therapeutic agents [22]. All of the four inhibitors as well as GC376 and the ligand Z369936976 have hydrophobic or hydrogen bond interactions with the essential catalytic residues His^41^ and Cys^145^ of Mpro. Inhibitor GC376 has a peptide bond. Similar to the four selected inhibitors, CB-25, CP-1 and LC24-20 also have a peptide bond in each of them. GC376 and CB21, while having a relatively lower IC50, have 15 hydrogen bond or hydrophobic interactions with Mpro while LC24-20, with a higher IC50, has 19 hydrogen bond or hydrophobic interactions with Mpro. It seems that having more interactions does not correlate with a lower IC50, as each interaction can contribute a different free energy. Compared with GC376, both CB-21 and LC24-20 have interactions with 12 of the same amino acids. Among them, 11 amino acids were the same and only 1 amino acid was different: GC376 and CB-21 had interactions with Phe^140^ while GC376 and LC24-20 had interactions with Pro^168^. Besides the 12 same amino acids, GC376 had interactions with Pro^168^, His^172^ and Gln^192^ with Mpro while CB-21 had interactions with Thr^25^, Thr^26^ and Leu^27^, possibility leading to the IC50 difference.

As SARS-CoV-2 mutates rapidly, to prioritize global monitoring and research, WHO, in collaboration with experts, national authorities, institutions and researchers, characterized specific Variants of Interest (VOIs) and Variants of Concern (VOCs), including the variants alpha, beta, delta and omicron. We searched the data from GISAID (https://gisaid.org/ (accessed on 23 January 2023)) and Nextstrain (https://nextstrain.org/ (accessed on 23 January 2023)) with 2910 SARS-CoV-2 genomes samples in the past six months (updated on 23 January 2023) [24]. Normalized Shannon entropy per codon for the whole genome was calculated, including Mpro (supplementary file). Results show that the main protease has 64 codon diversity entropies among the Mpro’s 306 amino acids. Not surprisingly, 74.6% of the codon diversity entropies equals to or is lower than 0.01. The two essential catalytic residues His^41^ and Cys^145^ are also reserved. Only four amino acids (1.3%) have a diversity entropy over 0.05: Gly^15^ (entropy 0.159), Leu^89^ (entropy 0.119), Lys^90^ (entropy 0.1) and Pro^132^ (entropy 0.584). However, these four amino acids are more than 10 Å from the catalytic center of Mpro. Therefore, the influence of mutations for Mpro activity and inhibition by our compounds are relatively low. On the contrary, 94 out of 1273 amino acids (7.4%) of SARS-CoV-2 spike protein have a diversity entropy over 0.05 (supplementary file). At the same time, 27 out of 1273 amino acids (2.1%) of spike protein have a diversity entropy over 0.5. Therefore, compared with inhibitors targeting spike protein, inhibitors targeting Mpro should be much more conserved. 

## 5. Conclusions

In this study, we evaluated four novel SARS-CoV-2 Mpro inhibitors with low IC50s and a high binding affinity, through a combined study of molecular modelling, docking, inhibition of enzyme activity, inhibitor binding, inhibitor toxicity and a ligand/enzyme interaction study. The new inhibitors have relatively higher bioavailabilities and lower toxicity compared with the well-known GC376 with high efficacy. They all have interactions with the essential catalytic residues His^41^ and Cys^145^, thus contributing to their inhibition efficacy. These inhibitors have potential to become drug candidates following tests with SARS-CoV-2 and animal models. The established combined computational and experimental study has brought potential drug candidates with good efficacy and low toxicity and will continue to be useful for the fight against COVID-19 as well as for new viral pandemics.

## Figures and Tables

**Table 1:** Docking score of selected compounds.

Name	Company	ID	Molecular Mass (Da)	ChemPLP Score
CB-21	Chembridge	25917626	489	98.08
CB-25	Chembridge	55464888	483	92.36
CP-1	ChemSpace	CSCS00026316954	298	81.19
LC24-20	Life Chemicals	F0514-4479	617	90.29

**Table 2:** Drug likeliness, pharmacokinetic and oral toxicity evaluations of selected compounds.

ID	Lipophilicity Consensus Log Po/w	Water Solubility (mol/L)	GI Absorption	BBB Permeability	P-gp Substrate	Bioavailability Score	LD_50_ (mg/kg)
GC376	−2.18	Soluble (8.47 × 10^−4^)	Low	No	Yes	0.17	300
CB-21	2.45	Poorly Soluble (3.00 × 10^−5^)	High	No	Yes	0.55	1600
CB-25	3.25	Poorly Soluble (4.00 × 10^−6^)	High	No	Yes	0.55	576
CP-1	2.75	Moderatery soluble (1.36 × 10^−4^)	High	Yes	No	0.55	1000
LC24-20	5.02	Insoluble (3.59 × 10^−8^)	Low	No	No	0.55	3000

Po/w: octanol/water partition coefficient, GI: Gastrointestinal, BBB: Blood Brain Barrier, 50%, P-gp: P-glycoprotein, LD_50_: 50% Lethal Dose.

**Table 3:** Hydrogen bond and hydrophobic interactions between each compound and main protease.

Compound	Hydrogen Bond Interaction	Hydrophobic Interaction
Z369936976	Gly^143^, His^163^	Thr^25^, Thr^26^, His^41^, Phe^140^, Leu^141^, Asn^142^, Cys^145^, His^164^, Glu^166^
GC376	Phe^140^, Gly^143^, Cys^145^, His^163^, Glu^166^	His^41^, Leu^141^, Asn^142^, Ser^144^, His^164^, Met^165^, Pro^168^, His^172^, Gln^189^, Gln^192^
CB-21		Thr^25^, Thr^26^, Leu^27^, His^41^, Phe^140^, Leu^141^, Asn^142^, Gly^143^, Ser^144^, Cys^145^, His^163^, His^164^, Met^165^, Glu^166^, Gln^189^
CB-25	His^41^	Thr^24^, Thr^26^, Phe^140^, Leu^141^, Asn^142^, Gly^143^, Cys^145^, His^163^, His^164^, Met^165^, Glu^166^, Pro^168^, Arg^188^, Gln^189^, Thr^190^
CP-1	His^41^, His^163^	Met^49^, Phe^140^, Leu^141^, Asn^142^, His^164^, Met^165^, Glu^166^, Arg^188^, Gln^189^, Thr^190^
LC24-20		Thr^24^, Thr^25^, His^41^, Cys^44^, Thr^45^, Ser^46^, Met^49^, Leu^141^, Asn^142^, Gly^143^, Ser^144^, Cys^145^, His^163^, His^164^, Met^165^, Glu^166^, Pro^168^, Arg^188^, Gln^189^

## Supplementary Materials

The following supporting information can be downloaded at: https://www.mdpi.com/article/10.3390/v15020580/s1. Table S1: Normalized Shannon entropy per codon for SARS-CoV-2 Mpro and Spike protein.
